# Association of HLA-A*02:01 type with efficacy and toxicity of immune checkpoint inhibitor therapy in melanoma patients: a retrospective cohort study

**DOI:** 10.1186/s12885-025-13857-y

**Published:** 2025-03-28

**Authors:** Isabel Manger, Christina Schmitt, Carola Berking, Lars E. French, Julio Vera-Gonzalez, Lucie Heinzerling

**Affiliations:** 1 Department of Dermatology, Friedrich-Alexander-Universität Erlangen-Nürnberg (FAU), Uniklinikum Erlangen, CCC Erlangen-EMN, CCC WERA, Erlangen, Germany; 2 Department of Dermatology and Allergy, LMU University Hospital, LMU Munich, Munich, Germany; 3 Bavarian Centre for Cancer Research (BZKF), Uniklinikum Erlangen and LMU Munich, Munich, Germany; 4 Dr. Philip Frost, Department of Dermatology and Cutaneous Surgery, University of Miami Miller School of Medicine, Miami, FL USA; 5 Department of Dermatology, LMU University Hospital Munich, Frauenlobstr. 9-11, Munich, D-80337 Germany

**Keywords:** Predictive marker, IrAE, Risk factor, Immunotherapy, Genetic factors

## Abstract

**Background:**

Immune checkpoint inhibitors (ICI) are highly effective but may induce severe or even fatal and unpredictable immune-related adverse events (irAEs). It is unclear whether human leukocyte antigen (HLA) genes contribute to the susceptibility of developing irAEs during ICI therapy.

**Methods:**

This multicentre retrospective study investigated the association of irAE and outcome with HLA-A*02:01 status in a cohort of 97 patients with metastatic melanoma undergoing ICI therapy. Organ-specific irAEs and therapy outcome as assessed by response rate, progression-free survival (PFS) and overall survival (OS) were analysed depending on HLA type HLA–A*02:01. For the outcome only patients with cutaneous melanoma were analysed. Chi square test, exact fisher test, Kruskal Wallis test and log rank test were employed for statistical analysis (*p* ≤ 0.05).

**Results:**

The cohort included 38 HLA-A*02:01 positive (39.2%) and 59 HLA-A*02:01 negative (60.8%) patients. Data showed no evidence of an association of HLA-A*02:01 with organ-specific irAEs except for a numerical difference in immune-related colitis. Furthermore, response rates of the subgroup of patients with metastatic cutaneous melanoma did not differ between the two cohorts. The median PFS was 5 months and 8 months in HLA-A*02:01 positive and negative patients with cutaneous melanoma, respectively.

**Conclusion:**

HLA-A*02:01 was not associated with specific checkpoint inhibitor-induced organ toxicity in this cohort of HLA-A-typed melanoma patients. Interestingly, in the relatively small subgroup of patients with cutaneous melanoma an earlier progression in HLA-A*02:01 positive patients was observed, however not in the long term. These findings are exploratory due to the limited sample size and require validation in larger, prospective cohorts.

## Background

Although immune checkpoint inhibitors (ICI) substantially improve the outcomes of patients across different tumour entities, they also cause considerable toxicity with up to 59% of severe immune-related adverse events (irAEs) [1, 2]. These can occur in all organ systems, including the digestive tract (e.g. irColitis), the endocrine system (e.g. irThyreoiditis, irHypophysitis), skin (irDermatitis), lungs (irPneumonitis), and liver (irHepatitis). Up to 35.5% of cancer patients experience chronic irAE one year or more after discontinuing ICI-therapy [3]. IrAEs are fatal in 0.3–1.3% of patients [4, 5].

As ICI therapy has been increasingly administered in earlier tumour stages [6, 7], there is an urgent need to identify predictive factors for toxicity to effectively evaluate the benefit-risk ratio. Furthermore, identifying at-risk patients before initiating immunotherapy could not only help mitigate the severity of irAEs but also improve patient management. However, despite numerous studies investigating baseline predictive factors for irAEs, no marker has yet been successfully translated into clinical practice [8, 9]. Female sex has been found to increase the risk for irHepatitis and preexisting autoimmune diseases such as rheumatoid arthritis, psoriasis and polymyalgia rheumatica have been shown to increase the risk of cardiovascular events induced by ICI regardless of known cardiovascular risk factors [10, 11]. Low baseline interleukin-6 serum levels have been shown to increase the risk for irAEs, and an increase in interleukin (IL)-6 has been observed to precede irAEs [12, 13]. In addition, high absolute eosinophil counts at baseline in peripheral blood have been associated with an increased risk of irPneumonitis and anti-thyroid antibodies have been linked to irThyreoiditis [14, 15]. Furthermore, high levels of C-reactive protein, IL-6, CXCL5, CXCL9, CXCL10, and Ki-67 + regulatory T cells as well as Ki-67 + CD8 + T cells following the initiation of ICI therapy have been associated with the occurrence of irAEs [13, 16, 17].

On the other hand, an association between biomarkers and ICI response has been shown. An overrepresentation of interleukin (IL)-17 T helper (TH17) gene expression signatures (GES), as seen in BRAF-V600 mutated tumours, might lead to a better response to dual ICI therapy, but not to monotherapy. Thus, IL17 appears to play a role as a predictive biomarker for treatment outcome [18]. Additionally, low levels of eosinophil-cationic protein, eosinophilia and low signaling of exchange proteins activated by cAMP (EPAC) have been associated with a better prognosis in melanoma [19–21].

Human leukocyte antigen (HLA) molecules are pivotal in antigen presentation and T-cell-mediated immune activation. The diversity of HLA molecules, driven by polymorphisms, enables a broad range of antigen recognition but also contributes to variability in immune responses. This extraordinary diversity is further enhanced by high mutation rates and genetic recombination within the HLA system [22]. This diversity has profound implications for ICI-therapy since efficient presentation of tumour antigens by HLA class I molecules is essential for the CD8^+^ T-cell-dependent elimination of cancer cells. Specific HLA alleles, such as HLA-DRB1*11:01, have been linked to immune-related pruritus, and HLA-DQB1*03:01 has previously been shown to be associated with irColitis in metastatic melanoma and non-small cell lung cancer (NSCLC) [23]. Additionally, HLA-DR15, B52, and Cw12 as well as HLA-DQ*06:02 have been identified as potential risk factors for irHypophysitis [24], while incidence of irHypophysitis was significantly lower in patients with HLA-DR*53 [25]. Furthermore, in patients with various malignancies, a significant association has been observed between HLA-DRB3*01:01 and immune-related thrombocytopenia, HLA-DPB1*04:02 and immune-related hypokalemia/hyponatremia, leukopenia, and anemia, as well as between HLA-A*26:01 and immune-related bilirubin elevation following ICI therapy [26]. However, the extent to which patient-specific HLA-I genotypes influence the response to ICIs, such as anti-programmed cell death protein 1 (anti-PD-1) or anti-cytotoxic T lymphocyte-associated protein 4 (anti-CTLA-4), remains an area of ongoing investigation [27]. Among these, HLA-A*02:01, one of the most common HLA alleles, has received limited attention, particularly regarding its impact on ICI-induced irAEs and treatment outcomes [28].

Research has shown that HLA-A*02:01 can present longer 15-mer peptides that are subsequently recognized by T-cells, challenging the previous assumption that only HLA-B molecules could bind such extended peptides. This underscores the crucial role of HLA-A*02:01 in T-cell immunity [29].

The HLA-A*02 supertype has been associated with improved overall survival (OS) and progression free survival (PFS) in patients with advanced lung cancer treated with ICIs [30, 31]. However, a smaller study in NSCLC patients found no correlation between HLA-A*02 and OS or PFS, suggesting that HLA-A*02 may not be a reliable prognostic marker [32]. Other studies in patients with NSCLC found no significant correlation between this HLA type and the development of irAEs [31–33]. To follow up on these results, we sought to investigate whether an association exists in metastatic melanoma patients undergoing ICI therapy. By exploring this relationship, we aimed to determine whether HLA-A*02:01 could serve as a predictive biomarker for treatment outcomes and its presence would potentially contribute to survival benefits in melanoma immunotherapy.

While HLA-A*02 alleles share common structural and functional characteristics, they exhibit subtle differences in peptide presentation and immune response, which may influence immunotherapy efficacy. Therefore, we focused specifically on HLA-A*02:01, as it is known to bind longer peptides that are subsequently effectively recognized by T cells, highlighting its pivotal role in T-cell-mediated anti-tumour response.

The aim of this study was to determine whether HLA-A*02:01 serves as a predictive biomarker for irAEs in patients undergoing ICI therapy and whether its presence is associated with clinical outcomes, including overall response rate (ORR), PFS, or OS as well as toxicity. By analysing a cohort of ICI-treated patients with metastatic melanoma, we sought to bridge the knowledge gap regarding the interplay between the HLA polymorphism HLA-A*02:01, toxicity, and therapeutic efficacy.

## Materials and methods

### Study design

This multicentre retrospective cohort study was approved by the local ethics committees of the Friedrich-Alexander-Universität Erlangen-Nürnberg, (No. 195_20B, No. 16-17-Br, No. 17-10-B) and the ethics committee of the Ludwig-Maximilians-Universität Munich (No. 20-1122). It was conducted according to the declaration of Helsinki.

### Settings and participants

Patients were enrolled at the skin cancer centres of the Uniklinikum Erlangen and the University Hospital Munich, Germany, after giving their informed written consent. The clinical data were documented in electronic patient files. HLA typing was performed utilizing the standard clinical care procedure, employing sequence-specific priming-polymerase chain reaction (SSP-PCR) in EDTA blood. In one of the participating centres, a substantial number of patients had already been tested for HLA-A*02:01 as part of a previous study, while in another centre, HLA typing was performed according to local standard protocol.

Inclusion criteria were diagnosis of metastatic melanoma, treatment with ICI (anti-CTLA–4 antibody ipilimumab, anti-PD-1 antibodies nivolumab or pembrolizumab, or a combination of anti-PD-1 and anti-CTLA-4 antibodies), and a follow-up of at least 6 months after initiation of ICI therapy. Patients received their initial ICI therapy between 2012 and 2022. HLA-A*02:01 positive and negative patients with metastatic melanoma undergoing ICI therapy were compared with regard to occurrence of irAE and tumour outcome. The patients received ICI therapy following standard procedures for each specific drug administration. The development of irAEs was monitored by clinical history and symptom assessment with grading according to the Common Terminology Criteria for Adverse Events (CTCAE V5.0). Upon suspicion of an irAE, a comprehensive examination was carried out to exclude other causes, including infections, disease progression, or secondary pathologies. 

Efficacy data was analysed only for the subgroup of patients with cutaneous melanoma. Tumour response was assessed using the response evaluation criteria in solid tumours (RECIST 1.1) to categorize complete response (CR), partial response (PR), stable disease (SD) and progressive disease (PD) and was carried out every 3 months. Imaging included cranial magnetic resonance tomography (MRT) and computed tomography (CT) of the neck, thorax and abdomen. OS and PFS were assessed from first application of ICI therapy. Melanoma of unknown primary and acrolentiginous melanoma were included in the investigated melanoma cohort.

### Statistical methods

Statistical analysis was performed using chi-square tests, Fisher’s exact test, Kruskal Wallis test, Cox regression, Kaplan Meier analysis and log rank test, with a significance level of *p* ≤ 0.05 as statistically significant (*) and *p* ≤ 0.01 as highly significant (**). The chi-square test was applied to compare the occurrence of irAEs between HLA-A*02:01 positive and HLA-A*02:01 negative patients. However, for irDiabetes mellitus, irPneumonitis, and immune-related adrenal insufficiency, the number of affected patients was too low for the chi-square test to be appropriate. Therefore, for these specific irAEs, we used Fisher’s exact test to ensure a statistically valid comparison. Graphs were generated using SPSS and PowerPoint.

## Results

This study investigated 97 patients with metastatic melanoma undergoing ICI therapy to characterize the association of irAEs and tumour outcome with the HLA type HLA-A*02:01. Among all patients, 38 (39.2%) were HLA type A*02:01, while 59 patients (60.8%) were HLA type A*02:01 negative. All patients underwent ICI therapy, with 27 (27.8%) receiving the CTLA-4-inhibitor ipilimumab, 21 (21.6%) a PD1-inhibitor (pembrolizumab or nivolumab), and 49 (50.5%) a combination of both as their first ICI therapy (Table 1). The median age of patients at ICI initiation was 61 years in both groups. For irAE analyses, patients with cutaneous, uveal and mucosal melanoma were included, for tumour outcome only patients with metastatic cutaneous melanoma stage lV (American Joint Committee on Cancer 8th Edition AJCC 2017) were included. In total, 62.7% of HLA-A*02:01 negative patients (37 of 59) developed irAEs, compared to 73.7% (28 out of 38) of HLA-A*02:01 positive patients. Regarding the organ-specific subtypes or irAE, endocrine irAEs occurred in 34.2% and 25.4% of the patients with and without HLA-A*02:01, respectively; irHepatitis appeared in 31.6% and 20.3% of patients with and without HLA-A*02:01, respectively, and irColitis in 15.8% and 23.7% of patients with and without HLA-A*02:01, respectively (Table 1).

**Table 1:** Patient characteristics. Percentages may not sum up to 100 due to rounding. anti-PD1: anti-programmed cell death 1 antibodies (pembrolizumab or nivolumab), anti-CTLA-4: anti-cytotoxic t-lymphocyte antigen 4 antibody (ipilimumab), or a combination of both (pembrolizumab or nivolumab + ipilimumab). irEndocrine: immune-related endocrine adverse events with the subtypes thyroid adverse events (irThyreoiditis), pituitary adverse events (irHypophysitis), diabetes (irDiabetes mellitus), and adrenal insufficiency. Other observed irAEs included digestive tract adverse events (irColitis), hepatic adverse events (irHepatitis) and pneumologic adverse events (irPneumonitis). Severity grading was performed using the Common Terminology Criteria for Adverse Events (CTCAE, v5.0). In case of more than one irAE, grading was conducted for the most severe irAE

*Patient characteristics*		
Cohorts	HLA-A*02:01 positive patients	HLA-A*02:01 negative patients
*N* (%)	38 (39.2)	59 (60.8)
Median age (range)	61 (50–72 years)	61 (47–75 years)
Gender: female n (%)-male n (%)	18 (47.4) – 20 (52.8)	29 (49.2) – 30 (50.8)
***Tumour entity***		
Cutaneous melanoma n (%) Mucosal melanoma n (%) Uveal melanoma n (%)	14 (36.8) 0 (0.0) 24 (63.2)	25 (42.4) 2 (3.4) 32 (54.2)
***BRAF mutation status***		
Wildtype n (%) V600E n (%) Unknown n (%)	14 (36.8) 3 (7.9) 21 (55.3)	27 (45.8) 7 (11.9) 25 (42.4)
***Primary stage (AJCC 2017)***		
IV n (%)	38 (100)	59 (100)
***ICI therapy***		
Anti-PD1 n(%) Anti-CTLA-4 n(%) Anti-PD1 + Anti-CTLA4 n(%)	9 (23.7) 9 (23.7) 20 (52.6)	12 (20.3) 18 (30.5) 29 (49.2)
Development of irAE n (%) More than 1 irAE n (%)	28/38 (73.7) 14/38 (36.8)	37/59 (62.7) 16/59 (27.1)
***Type of irAE***		
irEndocrine n (%) irThyreoiditis n (%) irHypophysitis n (%) irDiabetes mellitus n (%) Adrenal insufficiency n (%) irColitis n (%) irHepatitis n (%) irPneumonitis n (%) other irAE n (%)	13 (34.2) 5 (13.2) 6 (15.8) 2 (5.3) 1 (2.6) 6 (15.8) 12 (31.6) 3 (7.9) 11 (28.9)	15 (25.4) 8 (13.6) 8 (13.6) 0 (0.0) 2 (3.4) 14 (23.7) 12 (20.3) 5 (8.5) 13 (22.0)
***CTCAE° (related to irAE cases)***		
°1 or °2 n (%) °$\:\ge\:$3 n (%)	19/28 (67.9) 9/28 (32.1)	20/37 (54.1) 17/37 (45.9)
***Tumour outcome for patients with cutaneous melanoma***		
OS (months) PFS (months) (95% CI) ORR n (%) PD n (%) SD n (%) PR n (%) CR n (%)	NR^a^ 5 (0.0–16.8) 4 (28.6) 8 (57.1) 2 (14.3) 2 (14.3) 2 (14.3)	NR^a^ 8 (3.1–12.9) 2 (8.0) 12 (48.0) 11 (44.0) 0 (0.0) 2 (8.0)

^a^ NR: not reached. The median could not be reached since >50% were alive at data cut-off. Overall survival (OS), progression-free survival (PFS), overall response rate (ORR), progressive disease (PD), stable disease (SD), partial response (PR) and complete response (CR) according to the response evaluation criteria in solid tumours (RECIST 1.1)

### IrEndocrine

**Fig. 1:**
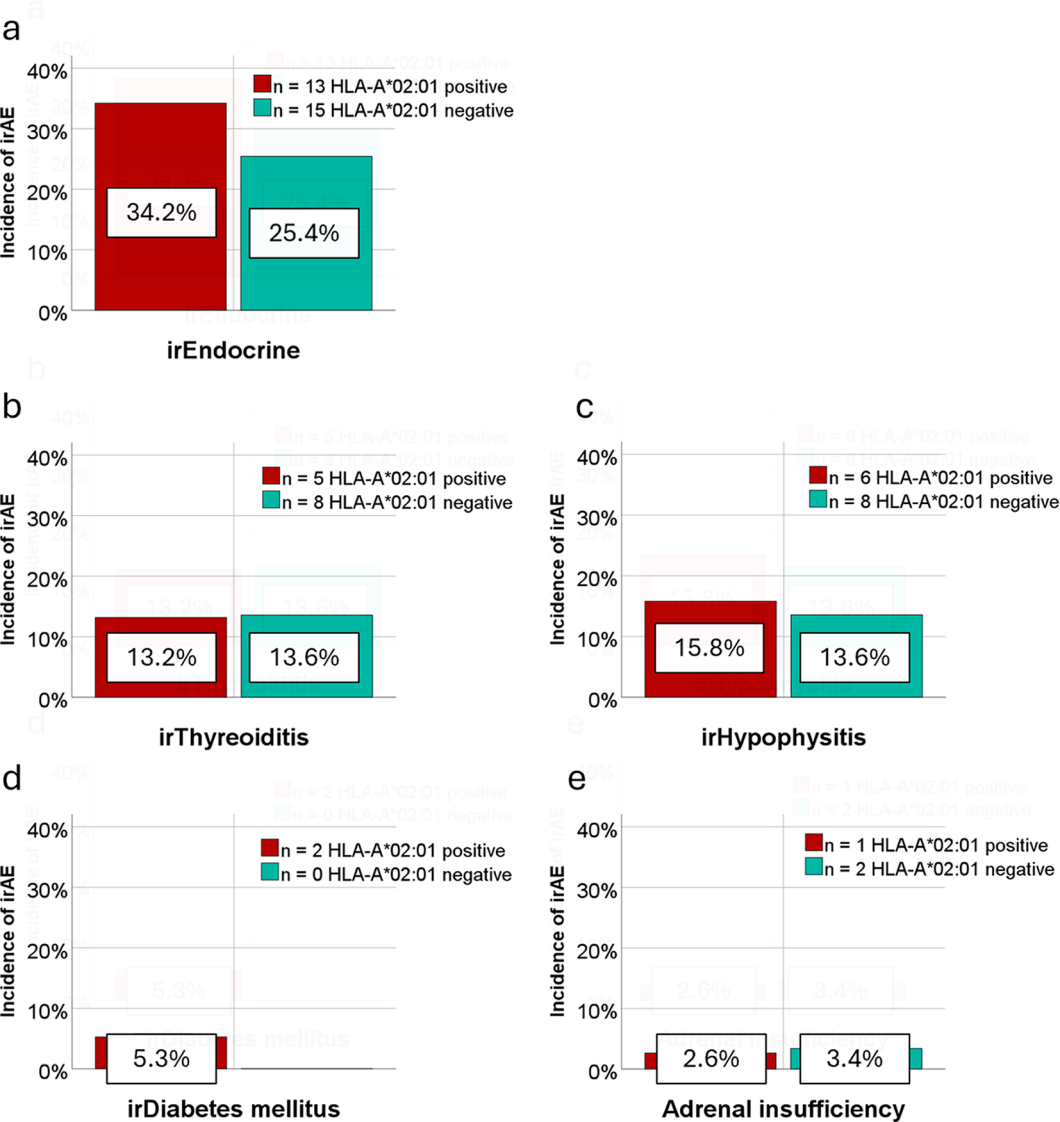
**a-e**: Incidence of endocrine irAEs and subtypes depending on HLA-A*02:01 status. In total, 97 patients with metastatic melanoma were investigated regarding the development of immune-related adverse events (irAEs) undergoing immune checkpoint inhibitor treatment. The proportion of each irAE subtype is displayed in relation to the number of HLA-A*02:01 positive and negative patients. (**a**) 28 patients developed an irEndocrine, 13 of these HLA-A*02:01 positive. Subtypes included (**b**) irThyreoiditis in 13 patients, (**c**) irHypophysitis in 14 patients, (**d**) irDiabetes mellitus in 2 patients, and (**e**) adrenal insufficiency in 3 patients. Abbreviations: immune-related adverse events (irAE), endocrinologic adverse events (irEndocrine), thyroid adverse events (irThyreoiditis), pituitary adverse events (irHypophysitis), diabetes (irDiabetes mellitus), adrenal insufficiency

Endocrine adverse events occurred in 28 of 97 patients (28.9%), 4 of these presented more than one endocrine irAE, including irThyreoiditis (*n* = 13), irHypophysitis (*n* = 14), irDiabetes mellitus (*n* = 2) and adrenal insufficiency (*n* = 3). Among HLA-A*02:01 positive patients, 34.2% developed endocrine adverse events compared to 25.4% of HLA-A*02:01 negative patients (Fig. 1a), however, this difference was not statistically significant. Thyroid-related adverse events appeared in 5 (13.2%) of 38 HLA-A*02:01 positive patients and 8 (13.5%) of 59 HLA-A*02:01 negative patients (Fig. 1b). Adverse events of the pituitary gland occurred in 6 (15.8%) and 8 (13.6%) of the HLA-A*02:01 positive and negative patients, respectively (Fig. 1c). Only 2 HLA-A*02:01 positive patients developed diabetes mellitus whereas none of the HLA-A*02:01 negative patients were affected (Fig. 1d). Immune-mediated adrenal insufficiency occurred in 1 (2.6%) HLA-A*02:01 positive patient and 2 (3.4%) HLA-A*02:01 negative patients (Fig. 1e).

### irColitis, irHepatitis, irPneumonitis

**Fig. 2:**
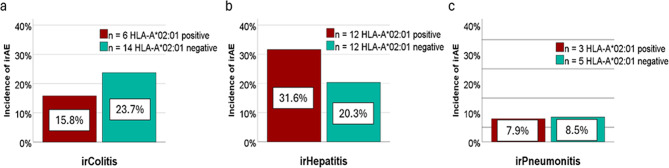
**a-c**: Incidence of organ-specific irAEs depending on HLA-A*02:01 status. In total, 97 patients with metastatic melanoma were investigated regarding the development of immune-related adverse events (irAEs) undergoing immune checkpoint inhibitor treatment, comparing HLA-A*02:01-positive and -negative patients. In total, (**a**) 20 patients developed irColitis, (**b**) 24 patients irHepatitis, and (**c**) 8 patients irPneumonitis

In total, 20 of 97 patients (20.6%) developed irColitis, with 15.8% (*n* = 6) of HLA-A*02:01 positive patients and 23.7% (*n* = 14) of HLA-A*02:01 negative patients affected. The difference was not statistically significant (Pearson chi-square = 0.345, Fig. 2a). Similarly, 24 of 97 patients (24.7%) developed irHepatitis, with 31.6% (*n* = 12) of HLA-A*02:01 positive patients and 20.3% (*n* = 12) of HLA-A*02:01 negative patients, also with no significant difference observed (Pearson chi-square = 0.210, Fig. 2b). Additionally, irPneumonitis occurred in 8 of 97 patients (8.2%), affecting 7.9% (*n* = 3) of HLA-A*02:01 positive patients and 8.5% (*n* = 5) of HLA-A*02:01 negative patients, again showing no statistically significant difference (Fig. 2c).

### Progression-free survival

**Fig. 3:**
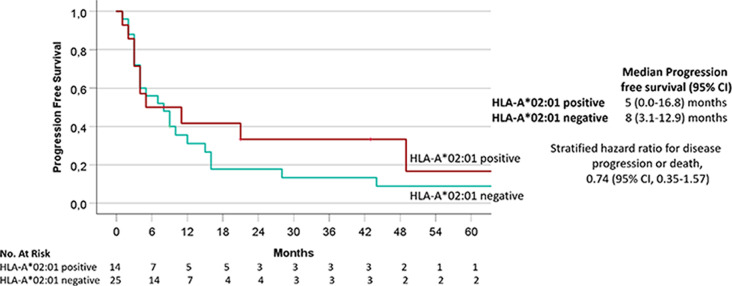
Progression-free survival (PFS) in months since the first ICI therapy of HLA-A*02:01 positive and negative patients with cutaneous melanoma. Kaplan-Meier analysis showed no significant difference comparing the HLA-A*02:01 positive (*n* = 14) and HLA-A*02:01 negative (*n* = 25) patient cohorts (*p* = 0.42)

A Kaplan-Meier analysis for PFS since the first initiation of ICI therapy was conducted in patients with cutaneous melanoma. For outcome evaluation only the subgroup of patients with metastatic cutaneous melanoma was analysed, as patients with uveal melanoma and mucosal melanoma are known to exhibit a poorer tumour response to ICI therapy compared to patients with cutaneous melanoma. The median follow-up was 24 months (range 2-117 months). The median PFS was 8 months and 5 months for HLA-A*02:01 negative and positive patients, respectively. The 6-months PFS was 56% for HLA-A*02:01 negative and 50% for HLA-A*02:01 positive patients. The 12-months PFS was 31.1% for HLA-A*02:01 negative and 41.7% for HLA-A*02:01 positive patients. The 36-months PFS was 13.3% for HLA-A*02:01 negative and 33.3% for HLA-A*02:01 positive patients. The differences were not statistically significant (*p* = 0.42, Fig. 3).

### Overall survival

**Fig. 4:**
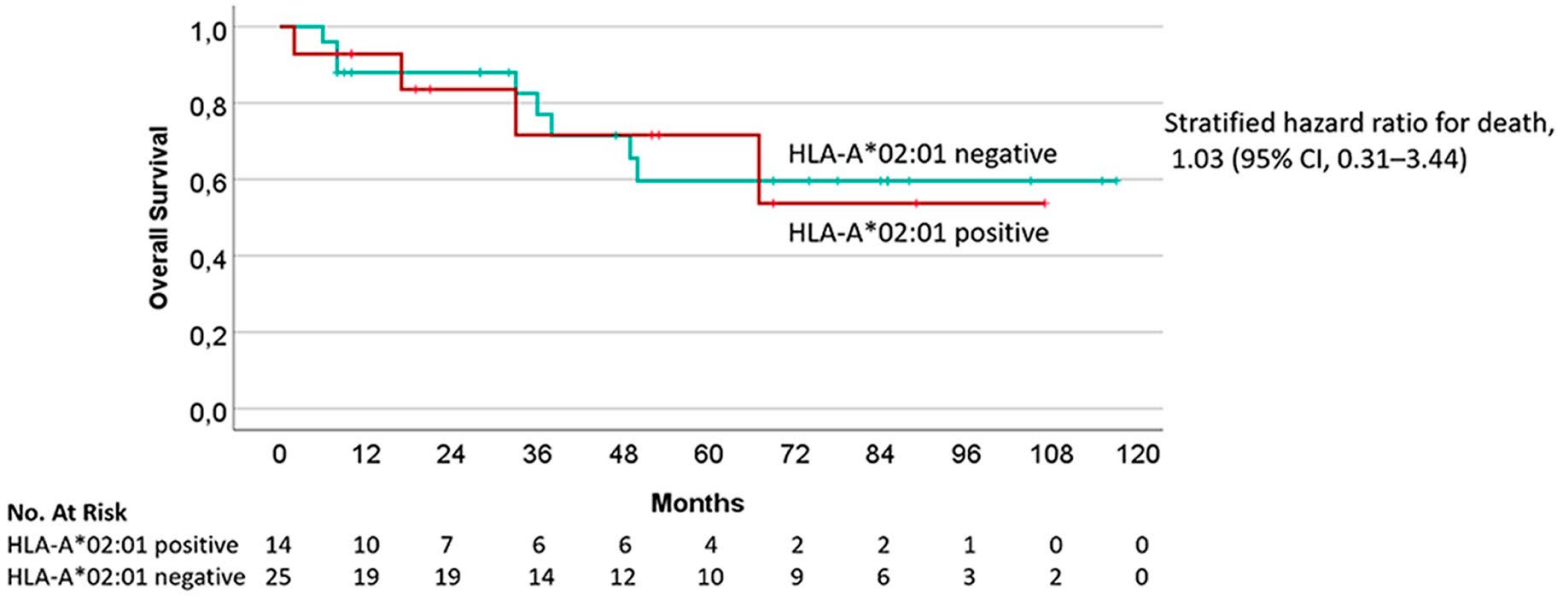
Overall survival (OS) in months since the first ICI-therapy of HLA-A*02:01 positive and negative patients with cutaneous melanoma. Kaplan-Meier analysis did not show significant OS differences comparing HLA-A*02:01 positive (*n* = 14) with negative (*n* = 25) patients (*p* = 0.96)

Kaplan-Meier analysis for OS since the first initiation of ICI therapy was assessed for patients with cutaneous melanoma only. The median follow-up was 24 months (range 2-117 months). Median OS was not reached in either group (Fig. 4). The difference between the two cohorts was not statistically significant (*p* = 0.96). The 84-months OS was 59.6% for HLA-A*02:01 negative and 53.7% for HLA-A*02:01 positive patients.

### Tumour response

Response rates of patients with cutaneous melanoma to the first ICI therapy were compared between the groups of HLA-A*02:01 positive patients (*n* = 14) and HLA-A*02:01 negative patients (*n* = 25). Response according to RECIST1.1 showed complete response (CR) in 4 (10.3%) patients with cutaneous melanoma, partial response (PR) in 2 (5.1%), stable disease (SD) in 13 (33.3%), and progressive disease (PD) in 20 patients (51.3%).

**Fig. 5:**
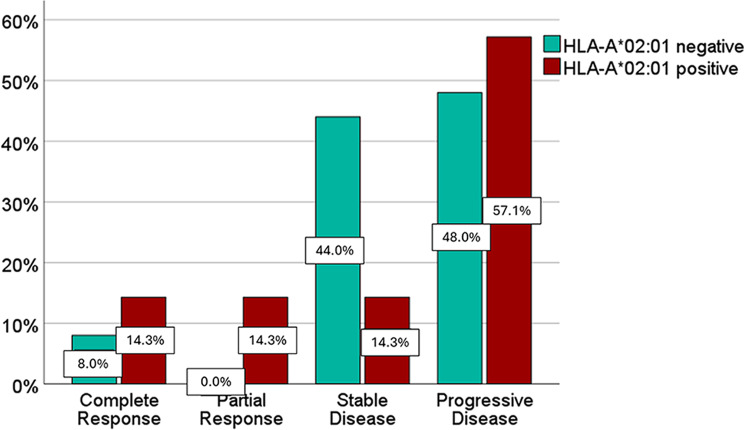
Best response to the first ICI therapy, comparing HLA-A*02:01 positive and negative status. Definition of the best response according to the Response Evaluation Criteria in Solid Tumours (RECIST 1.1): progressive disease (PD), stable disease (SD), partial response (PR), complete response (CR)

ORR in HLA-A*02:01 positive and negative patients was 28.6% and 8.0%, respectively. The difference was not statistically significant (*p* = 0.16). CR was observed in 2 HLA-A*02:01 positive patients (14.3%) and 2 HLA-A*02:01 negative patients (8.0%); PR was found in 2 HLA-A*02:01 positive patients (14.3%) and none of the HLA-A*02:01 negative patients (0.0%). SD was observed in 2 HLA-A*02:01 positive patients (14.3%) and 11 HLA-A*02:01 negative patients (44.0%). PD was observed in 8 HLA-A*02:01 positive patients (57.1%) and 12 HLA-A*02:01 negative patients (48.0%) (Fig. 5). Overall, we did not find a significant difference regarding the response rates (*p* = 0.95).

## Discussion

As of now, no baseline predictive marker exists to determine the risk of irAE occurrence. However, this would be of utmost importance since ICI therapy often induces severe irAEs, potentially leading to permanent sequelae with a significant reduction of quality of life or even fatal outcomes [3, 4, 34, 35]. Interestingly, HLA class II positivity has been suggested to influence susceptibility to immune evasion mechanisms in melanoma through neoantigen development [36]. Since ICI therapy may lead to a loss of tolerance against self-antigens, we hypothesized that HLA type could correlate with the occurrence of irAEs.

HLA polymorphisms influence the immune response by determining which peptides are presented by HLA molecules, thereby triggering distinct immune reactions [37, 38]. For example, the HLA-B*27 polymorphism is strongly associated with ankylosing spondylitis, and HLA-DR*4 is a known risk factor for rheumatoid arthritis [39, 40]. Moreover, certain HLA polymorphisms have been linked to specific tumour entities [41]. HLA polymorphisms influence not only antigen presentation but also the tumour microenvironment, thereby shaping the immune response against tumours [42]. The HLA-A*02, B*62 supertype may act as a favourable prognostic factor for immunotherapy in patients with NSCLC, as it is associated with a PD-L1-positive tumour microenvironment [43]. Tumour antigens, such as neoantigens arising from mutations, are presented more effectively by certain HLA alleles, such as HLA-A*03:01, which has been shown to present the mKRAS G12V peptide [44, 45]. Without this presentation, tumours may escape immune surveillance [46]. Recent findings suggest that the HLA tumour-antigen presentation score (HAPS), which integrates neoantigen binding affinity to HLA-I and allele divergence, correlates with improved survival in patients treated with ICIs, further underscoring the critical role of HLA-I-mediated antigen presentation in cancer immunotherapy [46].

HLA-G, a non-classical class I major histocompatibility complex (MHC) molecule, represents a key immune checkpoint that contributes to an immunosuppressive tumour microenvironment, facilitating immune evasion by tumour cells. HLA-G-based therapies, such as CAR-NK cells targeting HLA-G, show promise in overcoming tumour immune evasion. Considering that HLA-G is restricted to immune-privileged tissues and is nearly undetectable in normal cells, this could further reduce the harmful effects of HLA-G chimeric antigen receptor natural killer cell (CAR-NK) on normal tissue, a factor being increasingly investigated in preclinical studies [47].

In this study, HLA-A*02:01 was investigated as a potential predictive marker for irAEs of ICI therapy. Previous work has demonstrated an association of genetic expressions, as of a variant of the IL-7 gene, with an increased risk of irAE development in melanoma patients undergoing ICI therapy [48]. Specific subtypes of organ toxicities have been suggested to be associated with specific HLA type. For example, there might be a correlation between germline expression of HLA-B*35, HLA-DRB1*11 and the rate of irPneumonitis [23, 24, 49].

This study did not find a significant risk association between the presence of HLA-A*02:01 and ICI-induced irHepatitis or endocrine irAEs. Nevertheless, irColitis appeared in 7.9% more cases in the HLA-A*02:01 negative group compared to the HLA-A*02:01 positive group, although this difference was not statistically significant. This observation warrants further investigation to clarify whether HLA-A*02:01 status influences the development of specific irAEs.

Since little is known about the association of HLA-A*02:01 with the tumour outcome, we investigated this HLA subtype for its potential impact on response, PFS and OS in patients with metastatic cutaneous melanoma stage lV (AJCC 2017). Only patients with cutaneous melanoma were included in the efficacy analyses including tumour response, PFS, and OS, as metastatic uveal melanoma is known to have a poorer response to ICI therapy. The differences in PFS (*p* = 0.42) and OS (*p* = 0.96) between HLA-A*02:01 positive and negative patients were not statistically significant. The median PFS was 8 months and 5 months for HLA-A*02:01 negative and positive patients. The 84-months OS was 59.6% for HLA-A*02:01 negative and 53.7% for HLA-A*02:01 positive patients with metastatic cutaneous melanoma. These findings should be interpreted with caution and warrant validation in larger, prospective cohorts.

A known predictive marker for poorer PFS and OS under ICI therapy is HLA-A*03 [50]. We are currently not able to provide a pathophysiological rationale for the discrepancy between the findings with HLA-A*03 and our own results. Additionally, the presence of the HLA-DRB4 genotype has been shown to correlate with improved OS in metastatic NSCLC patients [51]. Further biomarkers are needed to evaluate decisions regarding the initiation of ICI therapy. Interestingly, not only the HLA-type but also the heterozygosity of the HLA-l loci (“A”, “B”, “C”) could play a role in the immune response. Maximized heterozygosity has been associated with improved OS under ICI therapy compared to patients with at least one homozygous HLA locus [27]. Studies indicate that higher heterozygosity at HLA-I loci, such as “A,” “B,” and “C,” increases the diversity of peptides presented to T-cells, thereby enhancing the potential diversity of immune responses against tumours. In contrast, patients with homozygous HLA loci may have reduced antigen presentation capacity, which could impair tumour immunosurveillance and response to immunotherapy [27, 30, 52]. Findings on the association between HLA heterozygosity/homozygosity and irAEs have been inconsistent across studies. While one study found no significant correlation [26], a study in patients with NSCLC reported an association between HLA-I homozygosity and a reduced risk of developing irAEs [33]. However, the occurrence of irAEs following ICI therapy showed no significant difference between the HLA-homozygous and HLA-heterozygous groups in patients with various other malignancies [26]. Nevertheless, HLA homozygosity was significantly linked to the development of irHepatitis in a study of 95 melanoma patients with ICI therapy [53]. While heterozygosity data were not collected in this cohort, future studies should explore its potential impact on the treatment outcomes, as it may represent a valuable biomarker for optimizing ICI therapy. Additionally, prospective analyses should complement retrospective findings to address the limitations of this study, including the small sample size and lack of heterozygosity data.

Previous studies have investigated the role of HLA-A*02 in cancer immunotherapy, with some reporting an association with improved OS in NSCLC patients, while others found no correlation with OS, PFS, or irAEs, highlighting inconsistencies in the existing data [30–33]. Nevertheless, to our knowledge, this is the first study to evaluate ICI-induced irAEs and tumour outcome in the context of the HLA-A*02:01 subtype. While no clear associations with organ-specific toxicities were observed, the trends identified in this small cohort should be considered exploratory and require validation in larger studies to ensure robust and generalizable conclusions. 

## Conclusions

In this cohort of HLA-typed melanoma patients, HLA-A*02:01 was not significantly associated with specific ICI-induced organ toxicity. Additionally, no statistically significant association was observed between HLA-A*02:01 status and disease progression or OS in patients with cutaneous melanoma. Further studies should include a larger number of patients, complete HLA-typing and analyses of heterozygosity to reflect the complexity of HLA-related immune responses. Identifying reliable markers could improve patient stratification, optimize risk-benefit assessments, and enhance personalized treatment strategies in immunotherapy.

## Data Availability

The raw data utilized in this study originate from electronic patient records of the Uniklinikum Erlangen and the LMU University Hospital Munich. These data have been anonymized and are stored in an Excel spreadsheet. Due to patient privacy concerns and institutional regulations, the datasets are not publicly available. However, the anonymized data can be obtained from the corresponding author upon reasonable request. Requests for access to the data should be directed to Lucie Heinzerling.

## Abbreviations

AJCC 2017: American Joint Committee on Cancer 8th Edition
CAR-NK: chimeric antigen receptor natural killer cell
CI: Confidence interval
CR: Complete response
CTCAE: V5.0 Common Terminology Criteria for Adverse Events version 5.0
CTLA4: Cytotoxic T-lymphocytee-associated protein 4
CT: Computed tomography
CXCL: CXC motif chemokine ligand
EPAC: Exchange proteins activated by cAMP
GES: Gene expression signatures
HAPS: HLA Tumour-antigen presentation score
HLA: Human leucocyte antigen
ICI: Immune-checkpoint inhibitor
IL: Interleukin
Ir: - Immune-related
irAE: Immune-related adverse event
MHC: Major histocompatibility complex
MRT: Magnetic resonance tomography
NR: Not reached
ORR: Overall response rate
OS: Overall survival
PD: Progressive disease
PD-1: Programmed cell death protein 1
PFS: Progression free survival
PR: Partial response
RESIST1.1: Response evaluation criteria in solid tumours 1.1
SD: Stable disease
SSP: -PCR Sequence-specific priming-polymerase chain reaction
TH: T helper
